# Hybrid Closed Loop Overcomes the Impact of Missed or Suboptimal Meal Boluses on Glucose Control in Children with Type 1 Diabetes Compared to Sensor-Augmented Pump Therapy

**DOI:** 10.1089/dia.2022.0518

**Published:** 2023-05-29

**Authors:** Régis Coutant, Elise Bismuth, Elisabeth Bonnemaison, Fabienne Dalla-Vale, Paul Morinais, Maelys Perrard, Jeanne Trely, Nathalie Faure, Natacha Bouhours-Nouet, Lucie Levaillant, Anne Farret, Caroline Storey, Aurélie Donzeau, Amélie Poidvin, Jessica Amsellem-Jager, Jérôme Place, Emmanuel Quemener, Jean François Hamel, Marc D. Breton, Nadia Tubiana-Rufi, Eric Renard

**Affiliations:** ^1^ Department of Pediatric Endocrinology and Diabetology, Angers University Hospital, Angers, France.; ^2^ Department of Pediatric Endocrinology and Diabetology, Robert Debré University Hospital, University of Paris, Paris, France.; ^3^ Department of Pediatrics, Tours University Hospital, Tours, France.; ^4^ Department of Pediatrics, Diabetes, Nutrition, Montpellier University Hospital, Montpellier, France.; ^5^ School of Medicine, Angers University Hospital, Angers, France.; ^6^ Department of Endocrinology, Diabetes, Nutrition, Montpellier University Hospital, Montpellier, France.; ^7^ Institute of Functional Genomics, University of Montpellier, CNRS, INSERM, Montpellier, France.; ^8^ Department of Biostatistics and Methodology, Angers University Hospital, Angers, France.; ^9^ Center for Diabetes Technology, University of Virginia, Charlottesville, Virginia, USA.; ^10^ INSERM Clinical Investigation Centre 1411, Montpellier, France.

**Keywords:** Children, Type 1 diabetes, Mismanagement, Meal bolus, Bolus omission, Hybrid closed loop

## Abstract

**Background:**

It is unclear whether hybrid closed-loop (HCL) therapy attenuates the metabolic impact of missed or suboptimal meal insulin bolus compared with sensor-augmented pump (SAP) therapy in children with type 1 diabetes in free-living conditions.

**Methods:**

This is an ancillary study from a multicenter randomized controlled trial that compared 24/7 HCL with evening and night (E/N) HCL for 36 weeks in children between 6 and 12 years old. In the present study, the 60 children from the E/N arm underwent a SAP phase, an E/N HCL for 18 weeks, then a 24/7 phase for 18 weeks, extended for 36 more weeks. The last 28–30 days of each of the four phases were analyzed according to meal bolus management (cumulated 6817 days). The primary endpoint was the percentage of time that the sensor glucose was in the target range (TIR, 70–180 mg/dL) according to the number of missed boluses per day.

**Findings:**

TIR was 54% ± 10% with SAP, 63% ± 7% with E/N HCL, and steadily 67% ± 7% with 24/7 HCL. From the SAP phase to 72 weeks of HCL, the percentage of days with at least one missed meal bolus increased from 12% to 22%. Estimated marginal (EM) mean TIR when no bolus was missed was 54% (95% confidence intervals [CI] 53–56) in SAP and it was 13% higher (95% CI 11–15) in the 24/7 HCL phase. EM mean TIR with 1 and ≥2 missed boluses/day was 49.5% (95% CI 46–52) and 45% (95% CI 39–51) in SAP, and it was 15% (95% CI 14–16) and 17% higher (95% CI 6–28), respectively, in the 24/7 HCL phase (*P* < 0.05 for all comparisons vs. SAP).

**Interpretation:**

HCL persistently improves glycemic control compared with SAP, even in case of meal bolus omission. ClinicalTrials.gov (NCT03739099)

## Introduction

Management of type 1 diabetes (T1D) is challenging in children, owing to suboptimal glucose monitoring and insulin treatment.^1–3^ Although meta-analyses support an association between adherence and metabolic control in pediatric T1D, adherence has been shown to be harder to achieve with the so-called intensive diabetes management.^4^ This is believed to be due to a mismatch between what scientists know is the best way to manage pediatric T1D and the degree to which children and their families can manage the disease.^4^ Bolus omission is common in children^5,6^ and is the leading cause of suboptimal glycemic control.^7^ Omission of two boluses per week results in a 0.5%–1% increase in HbA1c.^1,2,5–8^ Overall, most children and adolescents with T1D worldwide have mean HbA1c and sensor glucose above the recommended targets, suggesting that the burden of current optimal treatment may be too heavy for them.^9–11^

Hybrid closed-loop (HCL) systems, in which an algorithm automatically adjusts insulin delivery based on real-time sensor glucose levels, improve glycemic control in adolescents and children in free-living conditions over several weeks.^12–25^ In randomized parallel or crossover studies longer than 8 weeks, the mean improvement in time in range (TIR) (sensor glucose between 70 and 180 mg/dL) ranged from 10% to 15% and mean HbA1c decreased by 0.2%–0.5%.^12–25^ Of note, the mean HbA1c at enrollment was between 7.3% and 8.3%, indicating fair, that is, neither optimal not neglected-diabetes management in most of the participants.^12–25^

Of note, the algorithms in use are not conceived to handle unannounced meal boluses, although some use qualitative bolus announcement.^23,24^ The meal bolus management by the children and their families, namely missed boluses, delayed boluses after meal onset, and erroneous carbohydrate counting, was not reported in any of these studies, nor was their impact on glucose metrics in free-living conditions. It may be valuable to assess this impact, given that HCL systems are already available, whereas algorithms that deal with unannounced meals are still under investigation.^26–28^

The present study was motivated by the hypothesis that HCL could improve glucose control in T1D despite poor meal insulin management by kids in free-living conditions, thanks to the system-initiated corrections that could compensate defective meal insulin management. To test this hypothesis, we performed an ancillary study from a randomized 1:1 parallel HCL trial using Control-IQ system that started in November 2018 and included 120 children with T1D aged 6–12 years.^17^ The trial comprised a phase of sensor-augmented pump (SAP) therapy, an 18-week randomized phase of evening and night (E/N) compared with 24/7 HCL, an 18-week 24/7 HCL phase for all the subjects (hence 36 weeks of HCL), and followed by a 36-week 24/7 HCL extension phase (hence 72 weeks of HCL) to assess the efficacy and durability of the system in free-living conditions.^17^ Glucose metrics and insulin doses were collected throughout the trial.

In the present ancillary study, we additionally collected daily meal bolus management for 28–30-day periods at the end of each of the four phases described above in the 60 children initially randomized to the E/N group (cumulating a total of 6817 studied days). We analyzed (1) whether meal boluses were performed on time, delayed, or omitted; and (2) whether meal boluses were optimal for limiting the 1-h postprandial rise in sensor glucose below 200 mg/dL. This allowed us to compare the impact of meal insulin mismanagement on glucose metrics between SAP, E/N HCL, and 24/7 HCL phases.

## Materials and Methods

### Patients and trial conduct

The main trial was a prospective, open-label, randomized control trial conducted in four French University Hospitals, to compare 24/7 HCL with E/N HCL while using the Control-IQ system, including a t:slim X2 pump (Tandem Diabetes Care, San Diego, CA, USA), a G6 continuous glucose monitoring (CGM) device (Dexcom, San Diego, CA, USA), and a treat-to-target model predictive control algorithm. The criteria for inclusion in the study were as follows: prepubertal (Tanner score 1) children between 6 and 12 years old, with T1D for more than 1 year, plasma C peptide less than 66 pmoL/L and HbA1c less than 10% (<86 mmol/mol), and who had been treated by insulin pump for more than 6 months and were trained in carbohydrate counting.

Any associated chronic disease or therapy (except insulin) affecting glucose metabolism was an exclusion criterion. From November 2018 up to July 2019, 120 children were enrolled in this trial, underwent a 2-week run in SAP phase, and were randomly (1:1) allocated to 24/7 use of HCL or E/N HCL mode with daytime pump and CGM use for 18 weeks (comparative phase), then all the participants used 24/7 HCL for a further 18-week period. The complete trial design and the results have been published.^17^

### Ancillary study

For this ancillary study, only the 60 children who had been randomized into the E/N group were studied, out of the 120 children included in the main study, to compare the impact on glucose control of SAP, E/N, and HCL phases in the same patients according to meal insulin management (flowchart in Supplementary Fig. S1). Comparing the E/N and 24 HCL phases according to meal bolus management would give an insight into the algorithm's performance as a function of the use of the algorithm (no use in the SAP phase, nocturnal use in the E/N phase, 24-h use in the 24/7 HCL phase).

Of note, during the trial, the use of the Control-IQ software was temporarily suspended from March 8 to June 16, 2019, as a precaution after a software error was found during another study using this HCL system^15^: the included patients at that time (*n* = 50 in the E/N group) stopped using HCL, underwent a 10-week period of SAP therapy only, and the inclusion process was suspended during this period. We studied glucose control and meal insulin management during the SAP therapy of the software suspension period rather than during the initial 2-week run-in period because the included children were remarkably adherent during the run-in period with virtually no missed meal boluses.

Resolution of the software error allowed a full restart of the 18-week E/N and 24/7 HCL comparative periods for these patients and completion of the inclusion process through July 2019. From week 18 to 36, the patients randomized to E/N HCL moved to 24/7 HCL. After 36 weeks of the main trial, a 36-week extension phase was proposed.

Hospital visits were scheduled at study initiation and randomization, every 6 weeks after E/N HCL initiation until 18 weeks, and following the switch toward 24/7 HCL, every 9 weeks until 72 weeks. The visits consisted of physical examination, revision of pump parameters, downloading of pump and CGM data, and HbA1c measurements. Glucose metrics were collected for the whole SAP phase, the 18 weeks of the E/N HCL phase, the 18 weeks of the early 24/7 HCL phase, and the 36 weeks of the 24/7 HCL extension phase.

To specifically study meal insulin management, four periods of 28–30 days were analyzed in the present study, for a total of 6817 days: at the end of the SAP phase, at the end of 18-week E/N HCL phase, at the end of 18-week 24/7 HCL, and after further 36-week 24/7 HCL (hence at the end of 72 weeks of HCL use). The number of missed meal boluses per day (0, 1, 2, or more) and the number of suboptimal meal boluses per day (set as 0–1-h postprandial glucose ≥200 mg/dL or delayed boluses after the onset of the meal) (0, 1, 2, or more) were identified in each period of 28–30 days from the downloaded and computed pump and CGM data using the t:connect software from Tandem Diabetes.

The glucose metrics included the following: mean sensor glucose, percent of time above 180 mg/dL, percent of time between 70 and 180 mg/dL, and percent of time below 70 mg/dL. The insulin doses included the following: total daily insulin dose, system-initiated insulin dose (corresponding to basal insulin during the SAP phase), user-initiated meal insulin (according to estimated carbohydrate counting), user-initiated meal correction insulin (corresponding to the insulin dose that may be administered by the user to correct an inadequate sensor glucose value before the meal), and user-initiated correction insulin out of meal (corresponding to the insulin dose that may be administered by the user to correct an inadequate sensor glucose value out of meal). Only days with >90% data captured were analyzed, including mandatory daytime data capture for meal analyses.

#### Detection of missing or suboptimal meal boluses

Each day was analyzed independently by two observers (among J.T., M.P., and P.M.). In case of disagreement between the two observers, the discordant days were analyzed independently by two experienced diabetologists (R.C. and L.L.) to solve the discordance. Daily reports containing sensor data and daily glucose profile, insulin doses, and carbohydrate counts were analyzed using the following algorithm.

-when performed, the meal insulin was captured from the information on estimated carbohydrate counting: the number of daily meal boluses was mainly four in these children, sometimes three (when breakfast and/or afternoon snack were skipped)
-a missing meal bolus was defined as a bolus not performed at the expected time of meal (5:00–11:00 for the breakfast, 11:00–15:00 for the lunch, 15:00–18:00 for the afternoon snack, 18:00–23:00 for the dinner), associated with a sharp increase (in <1 h) in sensor glucose above 200 mg/dL and/or a sharp increase (in <1 h) in sensor glucose by >100 mg/dL with no previous hypoglycemia. For children and adults, the intake of 0.3 g/kg of carbohydrate (10 g on average in this age group) is expected to be associated with a mean increase in blood glucose of 55 mg/dL at 30 min.^29^
Therefore, these criteria should easily capture all the missing boluses. In a subgroup of 20 children who declared having missed at least one bolus on several specific days, this methodology appropriately captured all the cases of declared missed boluses. During the 24/7 HCL phases, a suspected missing bolus was associated with a contemporary sharp increase in system-initiated insulin, which made the detection of missed boluses easier.
- An optimal meal bolus was (arbitrarily) defined as a bolus associated with a postprandial (0–1 h) sensor glucose <200 mg/dL.
- A delayed meal bolus was defined as a bolus performed within 1 h of the onset of the rise in sensor glucose at the expected time of meal (and it was considered inappropriate). Some children and families used to order the insulin bolus at the end of the meal.
- A bolus performed more than 1 h after the rise in sensor glucose at the expected time of meal was considered a correction insulin out of meal (and likely corresponded to a missed meal bolus caught up later).

None of the children allocated to the E/N group dropped out of the study, and all continued to wear the HCL system.

### HCL algorithm

The control algorithm is based on model predictive control aiming to keep CGM within the 112.5–160 mg/dL range, and the closed-loop system requires user input for the management of meals.^15,17^ The algorithm adapts insulin delivery according to individual total daily dose and body weight after entering the usual pump settings, such as basal rate pattern, carbohydrate-to-insulin ratio, and correction factor. The administration of a meal bolus is followed by a refractory period of 60 min during which the algorithm does not change the preset basal rate: this was a precautionary process for limiting postprandial hypoglycemia that might be due to the additional effect of meal insulin and increased system-delivered insulin to counter the early glucose postprandial rise.

### Study outcomes

The primary outcome was the percentage of time that the sensor glucose level was in the TIR of 70–180 mg/dL (3.9–10.0 mmol/L). The secondary outcomes included the percentage of time that the sensor glucose level was below 70 mg/dL (3.9 mmol/L, time below range [TBR]); the mean daily sensor glucose level; and the HbA1c levels measured by high-performance liquid chromatography in blood samples. The total insulin daily dose (U/kg), the user-initiated meal insulin daily dose (U/kg), the user-initiated correction insulin (U/kg) (manual correction either at or out of mealtime), and the system-initiated daily dose (U/kg) were analyzed.

CGM data from each period of the 28–30 days were first used for the calculation of each metric, and then studied according to (1) the number of missed meal boluses per day and (2) the number of suboptimal or delayed meal boluses per day.

### Statistical analysis

Values are expressed as mean and standard deviation scores or 95% confidence intervals (CI), medians and 95% CI, and percentages. The TIR was compared between the last 28–30-day periods of each phase (SAP, E/N HCL, early 24/7 HCL, and late 24/7 HCL) with the use of a linear mixed-effects regression model. Mixed-effects models can give unbiased estimates of the means, when data are missing at random (here data of the SAP phase were missing in 10 of the 60 subjects, not yet included at the time of the software suspension). TIR was then compared between the phases according to the number of missed meal boluses per day or the number of suboptimal meal boluses per day (in this second analysis, only the days with no missed bolus were considered).

Phase and the number of missed meal boluses per day (0, 1, 2, or more) or the number of suboptimal meal boluses per day, set as 0–1-h postprandial glucose ≥200 mg/dL (0, 1, 2, or more), was considered fixed effects. All models included adjustment for repeated measurement in the same patients and clinical center (random effects). The covariance structure of the residuals was considered first-order autoregressive. Delayed boluses after meal onset were deemed suboptimal. Residual values were examined for homoscedasticity using scatterplots of residuals versus predicted values, and *q*–*q* plots. All the *P* values are two-sided at an alpha level of 0.05. Multiple testing was controlled by the weighted Bonferroni procedure.

Similar procedures were used for evaluating the impact of the phase and of the number of missed or suboptimal boluses on TBR, mean sensor glucose, and insulin doses. We used SPSS Statistics v25 (IBM Corp., Armonk, NY, USA) and STATA 14.2 (Stata Corp, College Station, TX, USA).

### Institutional review board approval

The study protocol was approved by the Comité de Protection des Personnes Nord Ouest IV, Lille, France, and the French National Agency for the Safety of Medicines and Health Products (ANSM) and was registered in ClinicalTrials.gov. All parents provided written consent, and all children gave their assent.

## Results

### Participants

The children (27 M, 33 F) were 9.3 ± 1.8 years old in the SAP phase and had had T1D for 5.8 ± 2.2 years. Patients' characteristics, glucose metrics, HbA1c levels, distribution of insulin doses, and the number of days with omitted and suboptimal meal insulin boluses according to the phase are presented in Table 1. Glucose metrics were similar between each full study phases and the last 28–30 days of each phase, during which meal bolus management was studied: only these 28–30-day periods are presented here. The percentage of daily time with sensor data was 96% (95% CI 94–98).

**Table 1.** Characteristics of the Patients and Glucose Metrics According to the Treatment Phase

Subjects (27 M/33 F)	SAP	E/N HCL, week 18	24/7 HCL, week 36	24/7 HCL, week 72
Age (years)	9.3 ± 1.8	9.6 ± 1.8	10.0 ± 1.8	10.7 ± 1.8
Weight (kg)	33.0 ± 8.1	34.6 ± 9.2	36.2 ± 9.4	39.5 ± 10.2
Height (cm)	135.1 ± 10.5	137.7 ± 11.4	139.9 ± 11.5	144.2 ± 11.8
HbA1c (%)	7.7 ± 0.7	7.3 ± 0.6^a^	7.2 ± 0.5^a^	7.3 ± 0.6^a^
Sensor glucose (mg/dL)	172 ± 19	166 ± 14^a^	161 ± 13^a,b^	161 ± 15^a,b^
Percent time with sensor glucose 70–180 mg/dL (TIR, %)	54 ± 10	63 ± 7^a^	67 ± 7^a,b^	67 ± 8^a,b^
Percent time with sensor glucose <70 mg/dL (TBR, %)	5 ± 3	3 ± 2^a^	3 ± 2^a^	3 ± 2^a^
Percent time with sensor glucose >180 mg/dL (TAR, %)	42 ± 10	34 ± 8^a^	31 ± 8^a,b^	31 ± 9^a,b^
Total insulin dose (IU/kg)	0.80 ± 0.14	0.84 ± 0.17	0.85 ± 0.16	0.86 ± 0.17
System-initiated insulin dose (IU/kg)	0.27 ± 0.07	0.37 ± 0.12	0.43 ± 0.14	0.44 ± 0.15
User-initiated meal bolus insulin (IU/kg)	0.43 ± 0.12	0.43 ± 0.15	0.41 ± 0.11	0.41 ± 0.12
User-initiated meal correction insulin (IU/kg)	0.05 ± 0.03	0.04 ± 0.03	0.01 ± 0.01	0.01 ± 0.01
User-initiated correction insulin out of meal (IU/kg)	0.04 ± 0.03	0.00 ± 0.00	0.00 ± 0.00	0.00 ± 0.00
Analyzed days (*N*)	1497	1738	1827	1755
No. of days with 0/1/≥2 omitted meal bolus(es)	1317/150/30	1477/226/35	1535/219/73	1404/281/105
No. of days with 0/1/≥2 suboptimal meal bolus(es)	303/395/619	237/428/812	322/476/737	365/435/618
No. of days with at least one delayed bolus	171	325	198	211

Demographics and HbA1c are measured at the end of each phase. Glucose metrics, insulin doses, and daily meal bolus management are reported for the last 28–30 days of each phase. Data are mean ± SD (medians and 95% CIs are reported in Supplementary Table S7). Percentages may not total 100 because of rounding. Mean HbA1c, mean sensor glucose, mean TIR, and mean TBR were compared between phases (mixed-effects model). TIR (70–180 mg/dL). TBR (<70 mg/dL). TAR (> 180 mg/dl). Suboptimal meal bolus: meal bolus that did not prevent a postprandial rise in sensor glucose above 200 mg/dL. For computing the number of days with suboptimal meal boluses, only the days with no missed boluses were considered.

^a^ Adjusted mean difference was significant (*P* < 0.0001) versus SAP phase.

^b^ Adjusted mean difference was significant (*P* < 0.0001) versus E/N HCL phase.

CI, confidence interval; E/N, evening and night; HCL, hybrid closed-loop; SAP, sensor-augmented pump; SD, standard deviation; TAR, time above range; TBR, time below range; TIR, time in range.

### Glucose control during the study phases

TIR was 54% ± 10% in the SAP phase, 63% ± 7% in the E/N HCL phase (*P* < 0.001 vs. SAP), 67% ± 7% in the early 24/7 HCL phase (36 weeks of HCL, *P* < 0.001 vs. SAP), and 67% ± 8% in the late 24/7 HCL phase (72 weeks of HCL), indicating a steady improvement with HCL. TIR was also 3% higher (95% CI 2–4, *P* < 0.0001) in 24/7 HCL compared with the E/N HCL phase.

HbA1c was 7.7% ± 0.7% in the SAP phase, 7.3% ± 0.6% in the E/N HCL phase, 7.2% ± 0.5% in the early 24/7 HCL phase, and 7.3% ± 0.6% in the late 24/7 HCL phase (*P* < 0.001 vs. SAP for all comparisons).

Since the glucose metrics were similar between the early and late 24/7 HCL phases, these two phases were grouped for mixed-model analyses (detailed in Supplementary Tables S1–S6).

### TIR and distribution of insulin doses according to the number of missed meal boluses

Over the SAP, the E/N HCL, the early 24/7 HCL, and the late 24/7 HCL phases, the percentage of days with at least one missed bolus increased from 12% to 15%, 16%, and 22%, respectively. Among the missed boluses, 20% were missed before breakfast, 23% before lunch, 42% before the afternoon snack, and 15% before dinner. The percentage of days with at least one missed bolus ranged from 1% to 84% per patient. All patients missed at least one meal bolus.

**FIG. 1.**
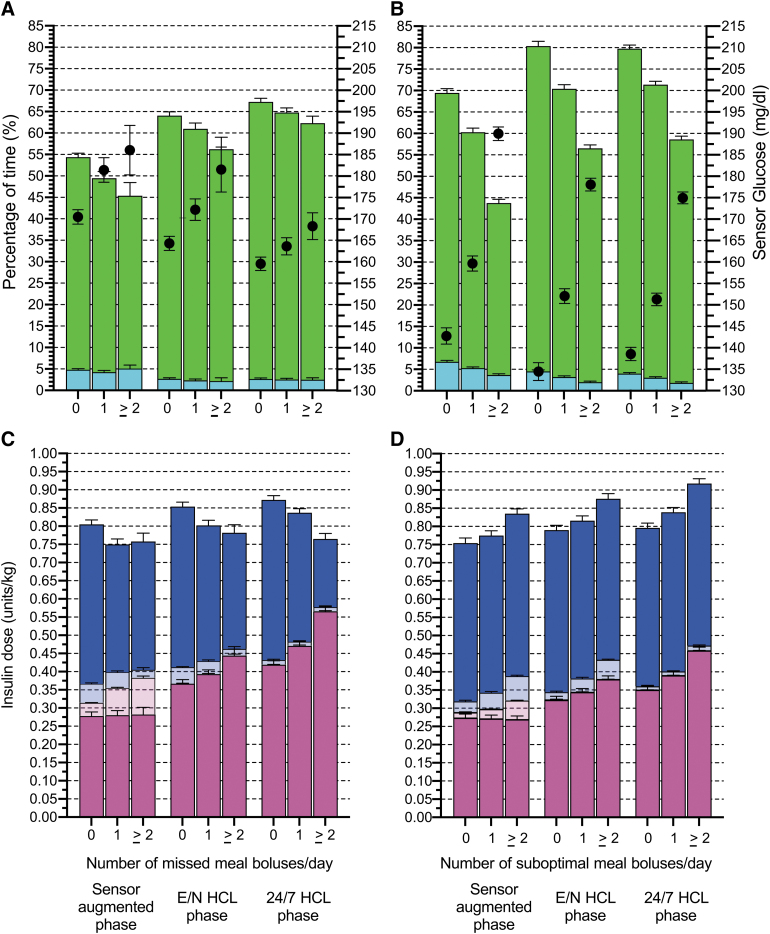
Glucose metrics and distribution of insulin doses in the SAP, evening/night, and 24/7 HCL phases according to the number of missed or suboptimal meal boluses. Percentage of time (%) for EM mean TIR (70–180 mg/dL, green bars) and TBR (<70 mg/dL, turquoise bars), and EM mean sensor glucose (mg/dL, black dots) according to the number of missed meal boluses **(A)**, and the number of suboptimal meal boluses (1-h postprandial glucose rise ≥200 mg/dL) **(B)**. TIR and TBR bars are superimposed (not stacked). Distribution of insulin doses (IU/[kg·day]) for EM mean system-initiated insulin or basal insulin for SAP phase (dark pink bars), user-initiated correction insulin out of meal (light pink bars), user-initiated correction insulin at meal (light blue bars), and user-initiated meal insulin (dark blue bars) according to the number of missed meal boluses **(C)** and the number of suboptimal meal boluses **(D)**. Insulin dose bars are stacked (not superimposed). Mixed-effects models and EM means are detailed in Supplementary Tables S1–S6. EM, estimated marginal; HCL, hybrid closed loop; SAP, sensor-augmented pump; TAR, time above range; TBR, time below range; TIR, time in range.

Glucose metrics and insulin doses (user-initiated and system-initiated insulin) according to the phase and number of missed meal bolus (0, 1, 2, or more) are presented in Figure 1A and Supplementary Table S3 (for comparisons between SAP, E/N, and 24/7 HCL phases).

When there was no bolus omission, the estimated marginal (EM) mean TIR was 54.4% (95% CI 52.6–56.2) in the SAP phase and 67.3 (95% CI 65.6–69.0) in the 24/7 HCL phase, with an adjusted mean difference of 12.9% (95% CI 11.1–14.6) (*P* < 0.0001).

When one bolus per day was omitted, the EM mean TIR was 49.5% (95% CI 46.4–52.5) in the SAP phase and 64.8 (95% CI 62.6–66.9) in the 24/7 HCL phase, with an adjusted mean difference of 15.3% (95% CI 14.4–16.2) (*P* < 0.0001).

Moreover, when two or more boluses per day were omitted, the EM mean TIR was 45.4% (95% CI 39.4–51.4) in the SAP phase and 62.3 (95% CI 59–65.6) in the 24/7 HCL phase, with an adjusted mean difference of 16.9% (95% CI 6.3–27.5) (*P* < 0.0001).

The metabolic consequences of omitting meal boluses were remarkably overcome by the HCL system compared with the SAP: TIR was 7.9% higher (95% CI 2.9–12.9, *P* < 0.0001) even when two or more boluses were omitted per day in the 24/7 HCL compared with TIR when all the boluses were delivered in the SAP phase.

During the SAP phase, the decrease in meal insulin associated with missed meal boluses was partly compensated by an increase in user-initiated correction insulin. During the HCL phases, the decrease in meal insulin associated with missed meal boluses was compensated by an increase of system-initiated insulin, whereas user-initiated correction insulin was negligible (Fig. 1C).

### TIR and distribution of insulin doses according to the number of suboptimal meal boluses per day

The percentage of days with more than one suboptimal bolus (meal boluses that did not prevent sensor glucose in the following hour from rising above 200 mg/dL, or delayed boluses after the onset of the meal) was 47%, 55%, 48%, and 44% in the SAP, E/N HCL, early 24/7 HCL, and late 24/7 HCL phases, respectively (Table 1). Glucose metrics (TIR, TBR, and mean sensor glucose) and insulin doses (user-initiated and system-initiated insulin) according to the phase and suboptimal bolus number are presented in Figure 1B and Supplementary Table S6 (for comparisons between SAP, E/N, and 24/7 HCL phases).

When the meal bolus assessment was optimal (no meal with a postprandial rise in glucose over 200 mg/dL), the EM mean TIR was 69.4% (95% CI 67.4–71.5) in the SAP phase and 79.8 (95% CI 78.1–81.4) in 24/7 HCL phase, with an adjusted mean difference of 10.3% (95% CI 7.3–13.4) (*P* < 0.0001).

At the other end, when the meal bolus assessment was poor (arbitrarily defined as two or more suboptimal meal boluses per day), the EM mean TIR was 43.8% (95% CI 42.1–45.5) in the SAP phase and 58.6 (95% CI 57.2–60.1) in the 24/7 HCL phase, with an adjusted mean difference of 14.9% (95% CI 12.7–17.0) (*P* < 0.0001).

The metabolic consequences of poor bolus assessment were attenuated but not overcome by the 24/7 HCL system: TIR was still 10.8% lower (95% CI 8.0–13.6) with 24/7 HCL with poor bolus assessment than with SAP therapy with optimal bolus assessment (calculation and timing).

During the SAP phase, suboptimal meal boluses were partly compensated by an increase in user-initiated correction insulin. During the HCL phases, suboptimal meal boluses were partially compensated by an increase in system-initiated insulin (Fig. 1D).

### Safety

One case of severe hypoglycemia occurred during the SAP phase. Neither ketoacidosis nor severe hypoglycemia occurred during the whole HCL phases.

## Discussion

This study in a population of children initially aged 6–12 years shows that HCL efficiently overcame the metabolic impact of meal bolus omission compared with SAP therapy, as EM mean TIR was 15% and 17% higher with 24/7 HCL compared with SAP therapy when one and two or more meal boluses per day, respectively, were missed. Remarkably, omitting two or more meal boluses per day with 24/7 HCL led to an EM mean TIR higher than the 54% EM mean TIR obtained when all boluses were delivered with SAP therapy. HCL also mitigated the metabolic consequences of poor (≥2 suboptimal meal boluses per day) bolus assessment (bolus calculation or delay in timing) compared with SAP therapy, as EM mean TIR was 59% with 24/7 HCL compared with 44% with SAP therapy.

Nevertheless, HCL with suboptimal boluses does not overcome SAP with optimal bolus assessment. In all cases, the best TIR was observed with 24/7 HCL, no missed bolus, and perfect bolus assessment (EM mean TIR 80%).

The TIR on 24/7 HCL observed here was similar to that reported both with the same HCL system^15,17^ and with another system in a population of similarly aged children.^25^ The original study by Renard et al. also showed that the greatest improvement was observed in children with the lowest TIR at enrollment,^17^ and this was also demonstrated in the study by Schoelwer et al.^30^ Similarly, in the study by Tauschmann et al. in children and adults with suboptimally controlled diabetes,^13^ those with the higher HbA1c and lower TIR at enrollment had the greatest improvement with HCL. This is confirmed in the present study, and further expended according to the number of missed meal boluses per day.

Although the current algorithms in use in HCL systems are not designed to remove the need for meal announcement, our study shows that they may efficiently attenuate the metabolic impact of unannounced meals. HCL also mitigated the metabolic consequences of poor bolus assessment, as the EM mean TIR was 15% higher with 24/7 HCL than with SAP therapy, while it was 10% higher with 24/7 HCL than with SAP therapy when all boluses were optimal. As expected, 24/7 HCL was associated with a significant reduction in user-initiated correction insulin. Whereas these corrections performed in the SAP phase were close to 0.1 U/(kg·day), thus limiting the metabolic impact of bolus omission, they became negligible in the HCL phases, suggesting a large decrease in treatment burden for children and families.

The results of the present study under free-living conditions were consistent with those of two short experimental studies in research centers.^31,32^ In a 2-day crossover trial in 16 adolescents, closed loop was compared with standard treatment for missed or underestimated meal insulin. Missing a snack (30 g of carbohydrates) insulin bolus or underestimating a lunch insulin bolus by 25% was associated with a 20%–25% lower TIR in the next 4 h with HCL compared with standard treatment.^31^ The apparently better performance of HCL in this experimental setting than that observed with the same system in free-living conditions was probably explained by the absence of correction insulin performed with standard treatment (as required by the study protocol), in contrast to what happened in real-life conditions in our study.

In another short experimental study in 12 adolescents, the TIR was 12% higher with closed loop compared with standard treatment when one meal bolus was missed, and one was suboptimal on the same day.^32^ Finally, one recent monocenter study in adults in free-living conditions using the control-IQ system identified 30 subjects with poor meal bolus announcement.^33^ In those with more than 90% of meal boluses missed (*n* = 10), the TIR after 1 year increased by 19% (95% CI 6–32) from baseline, consistent with the 17% (95% CI 6–27) increase found in our study.

Of note, during the HCL phase in our study, the EM mean TIR was 62% when at least two boluses per day were missed, whereas it was 59% when all the boluses were performed, but with at least two suboptimal boluses per day. This suggests that missing boluses had less metabolic impact than mis-assessing boluses in terms of calculation and timing. We believe that this apparent paradox can be explained by the 1-h refractory period following meal announcement. To avoid postprandial hypoglycemia due to excessive insulin delivery, the control IQ algorithm does not adjust insulin administration to sensor glucose values within 1 h of the meal announcement. In contrast, when the meal bolus is missed, the rise in sensor glucose is not analyzed by the algorithm as prandial, and it immediately increases insulin delivery to limit this rise.

The current study has some limitations. First, there was no control arm consisting of no use of the Control-IQ HCL system, as we compared the SAP and HCL phases in the same children in a predefined order. A previously reported randomized control trial using this system for several weeks already showed the efficacy and safety of the Control-IQ HCL system against SAP therapy.^15^ Remarkably, the TIR in the sensor-augmented (54% ± 10%) and the 24/7 HCL phases (67% ± 8%) in the present study was similar to those reported in the control and the 24/7 HCL groups of this 16-week randomized study, with a TIR of 55% ± 13% and 67% ± 10%, respectively.^15^ Second, we studied meal insulin management during the last 28–30 days of four phases, for a total of 6817 days, not during the entire duration of the phases.

However, we checked that the glucose metrics were similar between each entire period and the last 28–30 days. Thus, we believe that the results of the present study, specifically the attenuated effects of suboptimal or missed meal insulin boluses on glucose metrics by HCL, are valid. We did not specifically study glucose metrics for the 4–5-h period following completed or missed meal boluses, but only the 24-h glucose metrics: under free-living conditions, we had no information on estimated meal carbohydrate amounts in the event of a missed bolus, and it was difficult to determine the exact time of meal onset, and thus the time range to study.

Another limitation was the classification of boluses as missed or suboptimal. Although we took precautions to capture missed and suboptimal boluses, this relied on visual inspection of the daily sensor glucose and insulin profiles. Agreement between at least two observers was required to correctly classify boluses, but we could not formally eliminate the possibility that the observers could have made the same errors. Last, these children, initially aged 6–12 years, were likely less independent with boluses than older adolescents since diabetes management is usually supervised to a greater or lesser extent by parents. The consequences of missed and suboptimal boluses may be different in older HCL users, where the combination of physiological insulin resistance, suboptimal carbohydrate counting, and more autonomous management of diabetes (with less correction of bolus omission) may lead to differences in glucose metrics.

Finally, the sustainability of safety and efficacy results over 72 weeks in free-living conditions appears to complement previously reported data in clinical trials. However, the number of days with at least one missed bolus increased from 12% to 22% with time and HCL use. As this may be the consequence of the reduced burden of diabetes treatment, it suggests one caveat: maintaining safety skills may be paramount to expanding the use of HCL.

## Conclusion

In conclusion, our study showed that the best TIR is observed with 24/7 HCL when no bolus is missed or suboptimal. It also demonstrated that an HCL system in children aged 6–12 could efficiently mitigate poor meal insulin management, even in frequent omitted or suboptimal meal boluses. Provided that safety skills are properly maintained, this could support HCL use in a broad population of individuals with T1D.

## Data Sharing

The study protocol and statistical analysis plan for this project are available on request from the corresponding author. We will consider sharing data collected for the study, including deidentified individual participant data that underlie the results reported in this article on receipt of a request detailing the study hypothesis and statistical analysis plan. All requests should be sent to the corresponding author. The corresponding author and lead investigators of this study will discuss all requests and make decisions about whether data sharing is appropriate based on the scientific rigor of the proposal and after study sponsor approval. All applicants will be asked to sign a data access agreement.
