# Supplementary material for: Hybrid Closed Loop Overcomes the Impact of Missed or Suboptimal Meal Boluses on Glucose Control in Children with Type 1 Diabetes Compared to Sensor-Augmented Pump Therapy
Source: Diabetes Technol Ther. 2023 May 29;25(6):395–403. doi: 10.1089/dia.2022.0518 (PMC12352573; doi:10.1089/dia.2022.0518)
Supplement: Supplementary Figure S1 [file dia.2022.0518_suppl_figures1.docx]

**Supplemental File**

**Supplemental Figure 1 legend**

Flow chart and timeline of the ancillary study.

**Supplemental Figure 1**

Inclusion of 120 children aged 6-12 years with type 1 diabetes for more than 1 year and randomization to either 24/7 HCL or E/N HCL between November 2018 and July 2019 (main trial)

Software suspension for 10 weeks from 8^th^ March to 16^th^ June 2019. All the children included at that time (50 in the 24/7 HCL, 50 in the E/N HCL) returned to a sensor augmented phase using the same pump (Tandem t:slim) and the same sensor (Dexcom G6).

50 children already randomized to the E/N HCL underwent the SAP phase (ancillary study).

Downloading of the pump and sensor data (10 weeks).

Analyses of the last 28-30 days of the sensor augmented phase for diabetes management (n = 1497 days)

Restating of E/N HCL for 18 weeks for these 50 children

Completion of the inclusion / randomization (10 more children in the E/N group from June to July 2019)

=> 60 children on E/N HCL

Week 1 to week 18 of E/N HCL phase: downloading of the pump and sensor data

Analyses of the last 28-30 days of the E/N phase for diabetes management (n = 1738 days)

At the end of week 18 : switch to 24/7 HCL for 18 more weeks

Week 19 to week 36 of 24/7 HCL phase: downloading of the pump and sensor data

Analyses of the last 28-30 days of the 24/7 HCL phase for diabetes management (n = 1827 days)

Extension of the trial period on 24/7 HCL up to week 72

Downloading of the pump and sensor data every 9 weeks (week 36 to week 72)

Analyses of the last 28-30 days of the 24/7 HCL extension phase for diabetes management (n = 1755 days)
