# Supplementary material for: Hybrid Closed Loop Overcomes the Impact of Missed or Suboptimal Meal Boluses on Glucose Control in Children with Type 1 Diabetes Compared to Sensor-Augmented Pump Therapy
Source: Diabetes Technol Ther. 2023 May 29;25(6):395–403. doi: 10.1089/dia.2022.0518 (PMC12352573; doi:10.1089/dia.2022.0518)
Supplement: Supplementary Table S1 [file dia.2022.0518_suppl_tables1-s6.docx]

**Supplemental Table 1.** Mixed models for glucose metrics according to the four phases (sensor-augmented, E/N HCL, 24/7 HCL week 36, 24/7 HCL week 72) and the number of missed meal boluses (0, 1, 2 or more)

| **Parameter** | **TIR (%)** | **CI 95 %** | | **P** | **TBR (%)** | **CI 95 %** | | **P** | **Glucose (mg/dl)** | **CI 95 %** | | **P** |
| --- | --- | --- | --- | --- | --- | --- | --- | --- | --- | --- | --- | --- |
| **Constant*** | 54.4 | 52.6 | 56.2 | < 0.0001 | 4.8 | 4.2 | 5.3 | <0.0001 | 170.5 | 167.0 | 173.0 | <0.0001 |
| **Phase (Reference = SAP)** |  |  |  |  |  |  |  |  |  |  |  |  |
| **E/N HCL** | 9.6 | 8.4 | 10.8 | < 0.0001 | -2.1 | -2.4 | -1.8 | <0.0001 | -6.1 | -8.4 | -3.9 | <0.0001 |
| **24/7 HCL week 36** | 12.5 | 11.3 | 13.7 | < 0.0001 | -2.2 | -2.5 | -1.9 | <0.0001 | -10.5 | -12.7 | -8.2 | <0.0001 |
| **24/7 HCL week 72** | 13.2 | 12.0 | 14.4 | < 0.0001 | -2.1 | -2.4 | -1.7 | <0.0001 | -11.4 | -13.7 | -9.1 | <0.0001 |
| **Number of missed bolus if SAP**  **(Reference = 0 missed bolus)** |  |  |  |  |  |  |  |  |  |  |  |  |
| **1 missed bolus** | -5.0 | -7.7 | -2.2 | < 0.0001 | -0.5 | -1.3 | 0.2 | 0.142 | 11.0 | 5.9 | 16.2 | <0.0001 |
| **> 2 missed boluses** | -9.1 | -15.1 | -3.2 | 0.003 | 0.3 | -1.3 | 1.9 | 0.693 | 15.7 | 4.6 | 26.8 | <0.0001 |
| **Number of missed bolus if E/N HCL**  **(Reference = 0 missed bolus)** |  |  |  |  |  |  |  |  |  |  |  |  |
| **1 missed bolus** | -3.1 | -5.5 | -0.8 | 0.008 | -0.4 | -1.0 | 0.2 | 0.225 | 8.0 | 3.7 | 12.3 | <0.0001 |
| **> 2 missed boluses** | -8.1 | -13.5 | -2.7 | 0.003 | -0.5 | -1.9 | 0.9 | 0.485 | 17.6 | 7.6 | 27.7 | <0.0001 |
| **Number of missed bolus if 24/7 HCL week 36**  **(Reference = 0 missed bolus)** |  |  |  |  |  |  |  |  |  |  |  |  |
| **1 missed bolus** | -1.6 | -3.9 | 0.7 | 0.176 | -0.1 | -0.7 | 0.5 | 0.808 | 2.0 | -2.3 | 6.3 | 0.4 |
| **> 2 missed boluses** | -8.1 | -13.5 | -2.7 | 0.003 | -0.5 | -1.9 | 0.9 | 0.485 | 17.6 | 7.6 | 27.7 | <0.0001 |
| **Number of missed bolus if 24/7 HCL week 72**  **(Reference = 0 missed bolus)** |  |  |  |  |  |  |  |  |  |  |  |  |
| **1 missed bolus** | -3.5 | -5.6 | -1.3 | 0.001 | -0.2 | -0.8 | 0.3 | 0.438 | 6.1 | 2.1 | 10.0 | <0.01 |
| **> 2 missed boluses** | -3.5 | -7.1 | 0.2 | 0.061 | -0.4 | -1.4 | 0.5 | 0.401 | 5.4 | -1.3 | 12.2 | 0.1 |

* The constant corresponds to the predicted value of the outcome variable when the predictor variables equal zero. The predictor variables were the phases and the number of missed meal boluses per day. Reference categories were SAP phase and no missed bolus. The conditional effect of bolus omission by phase was calculated using linear combinations of the estimated model parameters, to allow a more intelligible presentation of the model results. The coefficients associated with each predictor variable denote the change in the predicted value of the outcome variable. The reference categories were the SAP phase, and no missed bolus per day. SAP sensor augmented pump therapy, E/N HCL evening and night hybrid closed loop, 24/7 HCL hybrid closed loop. TIR time in range. TBR time below range. Glucose = sensor glucose

**Supplemental Table 2** Mixed models for glucose metrics according to the three phases (sensor-augmented, E/N HCL, grouped 24/7 HCL) and the number of missed meal boluses (0, 1, 2 or more)

| **Parameter** | **TIR (%)** | **CI 95%** | | **P** | **TBR (%)** | **CI 95%** | | **P** | **Glucose (mg/dl)** | **CI 95%** | | **P** |
| --- | --- | --- | --- | --- | --- | --- | --- | --- | --- | --- | --- | --- |
| **Constant*** | 54.4 | 52.6 | 56.2 | < 0.0001 | 4.8 | 4.2 | 5.3 | < 0.0001 | 170.5 | 167.0 | 173.5 | <0.0001 |
| **Phase (Reference = SAP)** |  |  |  |  |  |  |  |  |  |  |  |  |
| **E/N HCL** | 9.6 | 8.4 | 10.8 | <0.0001 | -2.1 | -2.4 | -1.8 | <0.0001 | -6.1 | -8.4 | -3.9 | <0.0001 |
| **24/7 HCL** | 12.8 | 11.8 | 13.9 | <0.0001 | -2.1 | -2.4 | -1.8 | <0.0001 | -10.9 | -12.9 | -8.9 | <0.0001 |
| **Number of missed bolus if SAP**  **(Reference = 0 missed bolus)** |  |  |  |  |  |  |  |  |  |  |  |  |
| **1 missed bolus** | -5.0 | -7.7 | -2.2 | <0.0001 | -0.5 | -1.3 | 0.2 | 0.143 | 11.0 | 5.9 | 16.2 | <0.0001 |
| **> 2 missed boluses** | -9.1 | -15.1 | -3.2 | 0.003 | 0.3 | -1.3 | 1.9 | 0.695 | 15.7 | 4.6 | 26.8 | 0.006 |
| **Number of missed bolus if E/N HCL**  **(Reference = 0 missed bolus)** |  |  |  |  |  |  |  |  |  |  |  |  |
| **1 missed bolus** | -3.1 | -5.4 | -0.8 | 0.009 | -0.4 | -1.0 | 0.2 | 0.223 | 7.9 | 3.6 | 12.3 | <0.0001 |
| **> 2 missed boluses** | -8.0 | -13.4 | -2.6 | 0.003 | -0.5 | -1.9 | 0.9 | 0.487 | 17.5 | 7.5 | 27.6 | 0.001 |
| **Number of missed bolus if 24/7 HCL**  **(Reference = 0 missed bolus)** |  |  |  |  |  |  |  |  |  |  |  |  |
| **1 missed bolus** | -2.5 | -4.1 | -1.0 | 0.002 | -0.1 | -0.6 | 0.3 | 0.49 | 4.1 | 1.2 | 7.1 | 0.006 |
| **> 2 missed boluses** | -5.2 | -8.2 | -2.2 | 0.001 | -0.2 | -0.9 | 0.6 | 0.7 | 9.1 | 3.5 | 14.7 | 0.001 |

* The constant corresponds to the predicted value of the outcome variable when the predictor variables equal zero. The predictor variables were the phases and the number of missed meal boluses per day. Reference categories were SAP phase and no missed bolus. The conditional effect of bolus omission by phase was calculated using linear combinations of the estimated model parameters, to allow a more intelligible presentation of the model results. The coefficients associated with each predictor variable denote the change in the predicted value of the outcome variable. The reference categories were the SAP phase. and no missed bolus per day. SAP sensor augmented pump therapy. E/N HCL evening and night hybrid closed loop. 24/7 HCL hybrid closed loop. TIR time in range. TBR time below range. Glucose = sensor glucose

**Supplemental Table 3** Estimated marginal means from the mixed models for glucose metrics according to the three phases (sensor-augmented, E/N HCL, grouped 24/7 HCL) and the number of missed meal boluses (0, 1, 2 or more)

| **Glucose metrics** | **TIR (%)** | **CI 95 %** | | **TBR (%)** | **CI 95 %** | | **Glucose (mg/dl)** | **CI 95 %** | |
| --- | --- | --- | --- | --- | --- | --- | --- | --- | --- |
| **SAP and 0 missed bolus** | 54.4 | 52.6 | 56.2 | 4.8 | 4.3 | 5.3 | 170.5 | 167.1 | 173.8 |
| **SAP and 1 missed bolus** | 49.5^1^ | 46.4 | 52.5 | 4.3 | 3.5 | 5.1 | 181.4^1^ | 175.8 | 187.1 |
| **SAP and 2 or more missed boluses** | 45.4 | 39.4 | 51.4 | 5.1 | 3.5 | 6.7 | 186.1 | 174.8 | 197.4 |
| **E/N HCL and 0 missed bolus** | 64.1^2^ | 62.3 | 65.8 | 2.7^2^ | 2.2 | 3.2 | 164.3^2^ | 161.0 | 167.6 |
| **E/N HCL and 1 missed bolus** | 61.0^2^ | 58.3 | 63.6 | 2.3^2^ | 1.6 | 3.0 | 172.2^1^ | 167.3 | 177.1 |
| **E/N HCL and 2 or more missed boluses** | 56.2 | 50.7 | 61.7 | 2.2 | 0.7 | 3.6 | 181.5^1^ | 171.3 | 191.8 |
| **24/7 HCL and 0 missed bolus** | 67.3^2^ | 65.6 | 69.0 | 2.7^2^ | 2.2 | 3.1 | 159.6^2^ | 156.5 | 162.7 |
| **24/7 HCL and 1 missed bolus** | 64.8^2^ | 62.6 | 66.9 | 2.5^2^ | 2.0 | 3.1 | 163.7^2^ | 159.7 | 167.6 |
| **24/7 HCL and 2 or more missed boluses** | 62.3^1,2^ | 59.0 | 65.6 | 2.5 | 1.6 | 3.4 | 168.3 | 162.2 | 174.5 |

^1^ p< 0.05 vs. 0 missed bolus of the same phase. ^2^ p < 0.05 vs. the same bolus count of the SAP phase (weighted Bonferroni tests). SAP sensor augmented pump therapy, E/N HCL evening and night hybrid closed loop, 24/7 HCL hybrid closed loop. TIR time in range. TBR time below range. Glucose = sensor glucose

**Supplemental Table 4** Mixed models for glucose metrics according to the four phases (sensor-augmented, E/N HCL, 24/7 HCL week 36, 24/7 HCL week 72) and the number of suboptimal meal boluses (0, 1, 2 or more)

| **Parameter** | **TIR (%)** | **CI 95 %** | | **P** | **TBR (%)** | **CI 95 %** | | **P** | **Glucose (mg/dl)** | **CI 95 %** | | **P** |
| --- | --- | --- | --- | --- | --- | --- | --- | --- | --- | --- | --- | --- |
| **Constant*** | 69.4 | 67.4 | 71.5 | <0.0001 | 6.8 | 6.2 | 7.4 | <0.0001 | 142.8 | 139.0 | 146.5 | <0.0001 |
| **Phase (Reference = SAP)** |  | . | . | . |  | . | . | . |  | . | . | . |
| **E/N HCL** | 11.0 | 8.6 | 13.3 | <0.0001 | -2.2 | -2.9 | -1.5 | <0.0001 | -8.3 | -12.7 | -4.0 | <0.0001 |
| **24/7 HCL week 36** | 10.9 | 8.8 | 13.1 | <0.0001 | -2.7 | -3.4 | -2.0 | <0.0001 | -5.1 | -9.1 | -1.1 | 0.013 |
| **24/7 HCL week 72** | 9.7 | 7.6 | 11.8 | <0.0001 | -2.8 | -3.5 | -2.2 | <0.0001 | -3.4 | -7.3 | 0.5 | 0.089 |
| **Number of suboptimal bolus if SAP**  **(Reference = 0 suboptimal bolus)** |  | . | . | . |  | . | . | . |  | . | . | . |
| **1 suboptimal bolus** | -9.2 | -11.2 | -7.1 | <0.0001 | -1.5 | -2.1 | -0.9 | <0.0001 | 16.9 | 13.1 | 20.7 | <0.0001 |
| **> 2 suboptimal boluses** | -25.7 | -27.6 | -23.8 | <0.0001 | -3.1 | -3.6 | -2.5 | <0.0001 | 47.2 | 43.6 | 50.7 | <0.0001 |
| **Number of suboptimal bolus if E/N HCL**  **(Reference = 0 suboptimal bolus)** |  | . | . | . |  | . | . | . |  | . | . | . |
| **1 suboptimal bolus** | -10.0 | -12.2 | -7.8 | <0.0001 | -1.3 | -2.0 | -0.6 | <0.0001 | 17.6 | 13.5 | 21.7 | <0.0001 |
| **> 2 suboptimal boluses** | -23.9 | -26.0 | -21.9 | <0.0001 | -2.5 | -3.1 | -1.9 | <0.0001 | 43.7 | 39.9 | 47.5 | <0.0001 |
| **Number of suboptimal bolus if 24/7 HCL week 36**  **(Reference = 0 suboptimal bolus)** |  | . | . | . |  | . | . | . |  | . | . | . |
| **1 suboptimal bolus** | -9.2 | -11.1 | -7.2 | <0.0001 | -1.1 | -1.7 | -0.5 | <0.0001 | 13.9 | 10.3 | 17.5 | <0.0001 |
| **> 2 suboptimal boluses** | -21.7 | -23.5 | -19.9 | <0.0001 | -2.2 | -2.8 | -1.6 | <0.0001 | 36.8 | 33.4 | 40.2 | <0.0001 |
| **Number of suboptimal bolus if 24/7 HCL week 72**  **(Reference = 0 suboptimal bolus)** |  |  |  |  |  |  |  |  |  |  |  |  |
| **1 suboptimal bolus** | -7.6 | -9.6 | -5.7 | <0.0001 | -0.8 | -1.4 | -0.2 | 0.008 | 11.6 | 8.0 | 15.2 | <0.0001 |
| **> 2 suboptimal boluses** | -20.6 | -22.5 | -18.8 | <0.0001 | -2.1 | -2.7 | -1.5 | <0.0001 | 36.2 | 32.8 | 39.6 | <0.0001 |

* The constant corresponds to the predicted value of the outcome variable when the predictor variables equal zero. The predictor variables were the phases and the number of suboptimal meal boluses per day. Reference categories were SAP phase and no suboptimal bolus. The conditional effect of suboptimal bolus assessment by phase was calculated using linear combinations of the estimated model parameters. to allow a more intelligible presentation of the model results. The coefficients associated with each predictor variable denote the change in the predicted value of the outcome variable. The reference categories were the SAP phase. and no suboptimal bolus per day (all optimal). SAP sensor augmented pump therapy. E/N HCL evening and night hybrid closed loop. 24/7 HCL hybrid closed loop. TIR time in range. TBR time below range. Glucose = sensor glucose. Suboptimal meal bolus: meal bolus that did not prevent a postprandial rise in sensor glucose over 200 mg/dl

**Supplemental Table 5** Mixed models for glucose metrics according to the three phases (sensor-augmented. E/N HCL, grouped 24/7 HCL) and the number of suboptimal boluses (0, 1, 2 or more)

| **Parameter** | **TIR (%)** | **CI 95%** | | **P** | **TBR (%)** | **CI 95%** | | **P** | **Glucose (mg/dl)** | **CI 95%** | | **P** |
| --- | --- | --- | --- | --- | --- | --- | --- | --- | --- | --- | --- | --- |
| **Constant*** | 69.4 | 67.4 | 71.5 | <0.0001 | 6.8 | 6.2 | 7.4 | <0.0001 | 142.8 | 139.0 | 146.5 | <0.0001 |
| **Phase (Reference = SAP)** |  | . | . | . |  | . | . | . |  | . | . | . |
| **E/N HCL** | 11.0 | 8.6 | 13.3 | <0.0001 | -2.2 | -2.9 | -1.5 | <0.0001 | -8.3 | -12.7 | -3.9 | <0.0001 |
| **24/7 HCL** | 10.3 | 8.4 | 12.2 | <0.0001 | -2.8 | -3.3 | -2.2 | <0.0001 | -4.2 | -7.7 | -0.7 | 0.017 |
| **Number of suboptimal bolus if SAP**  **(Reference = 0 suboptimal bolus)** |  | . | . | . |  | . | . | . |  | . | . | . |
| **1 suboptimal bolus** | -9.2 | -11.2 | -7.1 | <0.0001 | -1.5 | -2.1 | -0.9 | <0.0001 | 16.9 | 13.1 | 20.7 | <0.0001 |
| **> 2 suboptimal boluses** | -25.7 | -27.6 | -23.8 | <0.0001 | -3.1 | -3.6 | -2.5 | <0.0001 | 47.2 | 43.6 | 50.7 | <0.0001 |
| **Number of suboptimal bolus if E/N HCL**  **(Reference = 0 suboptimal bolus)** |  | . | . | . |  | . | . | . |  | . | . | . |
| **1 suboptimal bolus** | -10.0 | -12.2 | -7.8 | <0.0001 | -1.3 | -2.0 | -0.6 | <0.0001 | 17.6 | 13.5 | 21.7 | <0.0001 |
| **> 2 suboptimal boluses** | -23.9 | -26.0 | -21.9 | <0.0001 | -2.5 | -3.1 | -1.9 | <0.0001 | 43.6 | 39.9 | 47.4 | <0.0001 |
| **Number of suboptimal bolus if 24/7 HCL**  **(Reference = 0 suboptimal bolus)** |  | . | . | . |  | . | . | . |  | . | . | . |
| **1 suboptimal bolus** | -8.4 | -9.7 | -7.0 | <0.0001 | -0.9 | -1.4 | -0.5 | <0.0001 | 12.7 | 10.2 | 15.3 | <0.0001 |
| **> 2 suboptimal boluses** | -21.1 | -22.4 | -19.8 | <0.0001 | -2.1 | -2.5 | -1.7 | <0.0001 | 36.4 | 34.0 | 38.8 | <0.0001 |

* The constant corresponds to the predicted value of the outcome variable when the predictor variables equal zero. The predictor variables were the phases and the number of suboptimal meal boluses per day. Reference categories were SAP phase and no suboptimal bolus. The conditional effect of suboptimal bolus assessment by phase was calculated using linear combinations of the estimated model parameters, to allow a more intelligible presentation of the model results. The coefficients associated with each predictor variable denote the change in the predicted value of the outcome variable. The reference categories were the SAP phase, and no suboptimal bolus per day (all optimal). SAP sensor augmented pump therapy, E/N HCL evening and night hybrid closed loop, 24/7 HCL hybrid closed loop. TIR time in range. TBR time below range. Glucose = sensor glucose. Suboptimal meal bolus : meal bolus that did not prevent a postprandial rise in sensor glucose over 200 mg/dl

**Supplemental Table 6** Estimated marginal means from the mixed models for glucose metrics according to the three phases (sensor-augmented, E/N HCL, grouped 24/7 HCL) and the number of suboptimal meal boluses (0, 1, 2 or more)

| **Glucose metrics** | **TIR** | **CI 95 %** | | **TBR** | **CI 95 %** | | **Glucose (mg/dl)** | **CI 95 %** | |
| --- | --- | --- | --- | --- | --- | --- | --- | --- | --- |
| **SAP and 0 suboptimal boluses** | 69.4 | 67.4 | 71.5 | 6.8 | 6.2 | 7.4 | 142.8 | 139.0 | 146.5 |
| **SAP augmented and 1 suboptimal bolus** | 60.3^1^ | 58.5 | 62.2 | 5.3^1^ | 4.7 | 5.9 | 159.7^1^ | 156.2 | 163.1 |
| **SAP augmented and 2 or more suboptimal boluses** | 43.8^1^ | 42.1 | 45.5 | 3.7^1^ | 3.2 | 4.2 | 189.9^1^ | 186.8 | 193.1 |
| **E/N HCL and 0 suboptimal boluses** | 80.4^2^ | 78.2 | 82.6 | 4.5^2^ | 3.9 | 5.2 | 134.5^2^ | 130.4 | 138.5 |
| **E/N HCL and 1 suboptimal bolus** | 70.4^1,2^ | 68.6 | 72.3 | 3.2^1,2^ | 2.6 | 3.8 | 152.1^1,2^ | 148.7 | 155.4 |
| **E/N HCL and 2 or more suboptimal boluses** | 56.5^1,2^ | 55.0 | 58.1 | 2.0^1, 2^ | 1.5 | 2.5 | 178.1^1,2^ | 175.2 | 181.0 |
| **24/7 HCL and 0 suboptimal boluses** | 79.8^2^ | 78.1 | 81.4 | 4.0^2^ | 3.5 | 4.5 | 138.6 | 135.5 | 141.6 |
| **24/7 HCL and 1 suboptimal bolus** | 71.4^1,2^ | 69.8 | 73.0 | 3.1^1,2^ | 2.6 | 3.6 | 151.3^1,2^ | 148.4 | 154.2 |
| **24/7 HCL and 2 or more suboptimal boluses** | 58.6^1,2^ | 57.2 | 60.1 | 1.9^1,2^ | 1.4 | 2.3 | 175.0^1,2^ | 172.2 | 177.7 |

^1^ p< 0.05 vs. 0 suboptimal bolus of the same phase. ^2^ p < 0.05 vs. the same bolus category of the SAP phase (weighted Bonferroni tests). SAP sensor augmented pump therapy, E/N HCL evening and night hybrid closed loop, 24/7 HCL hybrid closed loop. TIR time in range. TBR time below range. Glucose = sensor glucose. Suboptimal meal bolus : meal bolus that did not prevent a postprandial rise in sensor glucose over 200 mg/dl
