# Supplementary material for: Hybrid Closed Loop Overcomes the Impact of Missed or Suboptimal Meal Boluses on Glucose Control in Children with Type 1 Diabetes Compared to Sensor-Augmented Pump Therapy
Source: Diabetes Technol Ther. 2023 May 29;25(6):395–403. doi: 10.1089/dia.2022.0518 (PMC12352573; doi:10.1089/dia.2022.0518)
Supplement: Supplementary Table S7 [file dia.2022.0518_suppl_tables7.docx]

**Supplemental Table 7.** Glucose metrics (medians and 95% confidence interval) according to the treatment phase

| **Subjects (27 M / 33 F)** | **SAP** | **E/N HCL**  **week 18** | **24/7 HCL**  **week 36** | **24/7 HCL**  **week 72** |
| --- | --- | --- | --- | --- |
| Median sensor glucose (mg/dl) | 169 (138; 200) | 166 (141; 191) | 161 (143; 183) | 159 (139; 187) |
| Median percent time with sensor glucose 70-180 mg/dl (TIR, %) | 54 (38; 72) | 64 (51; 78) | 66 (53; 79) | 67 (52; 80) |
| Median percent time with sensor glucose < 70 mg/dl (TBR,%) | 4 (1 ; 11) | 2 (0 ; 8) | 2 (1 ; 6) | 2 (1 ; 6) |
| Median percent time with sensor glucose >180 mg/dl (TAR, %) | 41 (22 ; 56) | 34 (20 ; 47) | 31 (17 ; 46) | 29 (17 ; 46) |

Glucose metrics are reported for the last 28-30 days of each phase. Data are medians (95% confidence interval). Percentages may not total 100 because of rounding. SAP Sensor-augmented pump. HCL hybrid closed loop. E/N evening and night. TIR time in range (70-180 mg/dl). TBR time below range (< 70 mg/dl). TAR time above range (> 180 mg/dl).
